# Management of uveitic and chorioretinal conditions in pregnancy

**DOI:** 10.1038/s41433-025-04058-9

**Published:** 2025-11-07

**Authors:** Ariel Yuhan Ong, Evita Evangelia Christou, Christine A. Kiire, Naomi Primrose, William R. Tucker, Charlotte Frise, Samantha R. de Silva

**Affiliations:** 1 Oxford Eye Hospital, Oxford University Hospitals NHS Foundation Trust, Oxford, UK; 2 Moorfields Eye Hospital, Moorfields Eye Hospital NHS Foundation Trust, London, UK; 3 Institute of Ophthalmology, University College London, London, UK; 4 Queen Charlotte’s and Chelsea Hospital, Imperial College Healthcare NHS Trust, London, UK; 5 Nuffield Department of Clinical Neurosciences, University of Oxford, Oxford, UK

**Keywords:** Eye diseases, Quality of life

## Abstract

During pregnancy, women may develop de novo sight-threatening chorioretinal diseases or experience exacerbations of these conditions. Examples include macular neovascularisation, diabetic macular oedema, and posterior uveitis. Ophthalmologists may not necessarily recommend the standard treatment for these disorders when they arise in pregnant women due to a lack of evidence regarding safety and potential risks of teratogenicity and fetotoxicity, and a lack of experience with managing this patient population. However, withholding treatment may lead to irreversible maternal vision loss. In this review, we discuss therapies that may need to be considered in these conditions, including steroids, intraocular pressure-lowering treatment for steroid-induced glaucoma, systemic immunosuppressants, and intravitreal anti-vascular endothelial growth factor agents. We discuss the evidence base behind these treatments in terms of safety in pregnancy, bringing in the obstetric perspective. We also discuss common misconceptions surrounding different modes of delivery in pregnant women with chorioretinal disease.

## Introduction

Sight-threatening eye diseases may occasionally occur or worsen in pregnancy. Balancing the need to provide treatment to preserve maternal vision versus any potential risk to the fetus can be difficult, particularly as there is often a lack of data on the systemic effects of medications in pregnancy. Research in pregnant women has historically been challenging because of the complex physiology of this patient population, ethical concerns about intervening in a ‘vulnerable’ population, lack of regulatory support, and fears about fetal harm and attendant medico-legal liabilities [1–3]. The evidence base for managing ophthalmic conditions in this unique population is therefore relatively limited, and existing studies (especially animal studies) may be out of date, many having been conducted several decades ago.

One of the key concerns about managing these ophthalmic conditions in pregnant women is the fear of inducing obstetric complications, miscarriage, or teratogenicity. It is important to recognise that early pregnancy loss is common, even in healthy individuals—commonly cited as 10–20% of all pregnancies—and this is most often attributable to chromosomal abnormalities [4]. In addition, the overall risk of congenital malformations in the general population is estimated at 2–3% of all live births, though most malformations are relatively minor [5, 6]. Many women in whom ophthalmic therapy may need to be considered also have systemic comorbidities such as diabetes mellitus, hypertension, or autoimmune diseases, which drive their need for treatment in the first place, and which have a significant influence on pregnancy outcomes. Poorly controlled diabetes elevates the risk of early miscarriage and congenital malformations [7], while autoimmune diseases increase the risk of maternal morbidity and can also adversely impact placental function [8]. It is therefore important to be able to disentangle the inherent risk from their disease with the risk of treatment, which is not always easy or possible to do [9].

In this review, we aim to discuss some commonly encountered situations in the management of inflammatory and chorioretinal conditions in pregnancy, where treatment may be beneficial, and where multidisciplinary team collaboration between ophthalmologists and obstetricians/obstetric physicians is often helpful. This review focuses on the ophthalmic management of uveitic and chorioretinal conditions in pregnancy alone. Optimisation of systemic risk factors such as blood sugar and blood pressur, for diabetic and hypertensive retinopathy, is outside the scope of this review. We will consider the risks and benefits of potential treatments (including corticosteroids, systemic immunosuppressants, intravitreal anti-vascular endothelial growth factor (anti-VEGF) agents), interventions for potential side effects of some of these treatments such as intraocular pressure (IOP) rise, and modes of obstetric delivery in these women.

## Methods

PubMed was searched up to 1 March 2025 for relevant papers to inform the narrative review. A modular search strategy combining core concepts using Boolean operators was employed, the full details of which are provided in Supplementary S1. In brief, the search combined search terms for two key concepts: (1) pregnancy and gestation, and (2) pre-specified management domains (corticosteroids, IOP-lowering treatment, systemic immunosuppressants and intravitreal anti-VEGF). Separately, search terms for mode of obstetric delivery were combined with chorioretinal disease. No date restrictions were applied to the search. Reference lists of reviews and relevant studies were examined for other potentially relevant studies, and key guidelines were reviewed.

To ensure a comprehensive and clinically robust perspective, critical input was sought from a multidisciplinary team of specialists, including ophthalmologists with expertise in retinal and uveitic diseases, obstetricians, and obstetric physicians.

## Steroid treatment

Corticosteroids are commonly used in ophthalmic care for their broad anti-inflammatory and immunosuppressive properties. Theoretically, autoimmune conditions are expected to improve in pregnancy due to increased levels of circulating cortisol and other pregnancy-related changes in immunity [4]. This accords with findings from a small number of retrospective real-world studies, which suggest that the rates of new episodes of non-infectious uveitis remain the same or are elevated in the first trimester, decrease in the second and third trimester, and increase again soon after delivery before returning to pre-pregnancy levels [10–12].

The therapeutic approach to anterior or intermediate uveitis typically includes administration of topical steroid drops and/or intravitreal corticosteroid injections if concurrent cystoid macular oedema is present. Severe or recurrent anterior or intermediate uveitis, or cases of posterior uveitis, may require systemic corticosteroids or immunosuppression [13]. The pharmacokinetics of corticosteroid therapy may vary significantly depending on the route of administration (topical, intravitreal, periocular and systemic), the corticosteroid agent used, and individual patient characteristics [13, 14]. In particular, the different types of steroids have different effects on the fetus through differential placental metabolism.

Topical corticosteroids (e.g. prednisolone and dexamethasone eye drops) are effective in managing anterior segment inflammation and are generally considered to be safe in pregnancy. Teratogenicity following the use of topical corticosteroids has not been reported in humans. As in non-pregnant patients, there are risks of ocular side effects such as exacerbation of herpetic eye infections, steroid-induced IOP rise, and cataract formation. Eye drops may be systemically absorbed through the mucosal vessels and lead to low circulating levels, which could cross the placenta and reach the fetus [13, 15]. However, the patient can be reassured that the circulating levels are very low, and any medication that reaches the fetus will be at a far lower level than that known to cause any fetal complications, meaning that topical corticosteroids can be used as normal in pregnancy. Punctal occlusion—applying pressure over the nasolacrimal sac after instillation—can further minimise systemic absorption.

Treatment with intravitreal corticosteroids may be considered in some pregnant women with macular oedema associated with diabetes (diabetic macular oedema, DMO), retinal vein occlusion, or uveitis. Intravitreal corticosteroid agents differ in their characteristics. In particular, intravitreal triamcinolone does not achieve significant systemic serum levels and may therefore be considered safe in pregnancy [4]. Systemic dexamethasone doses from an intravitreal slow-release implant (Ozurdex®) are not thought to be high enough to be clinically relevant. Several case reports describe intravitreal triamcinolone and dexamethasone for the treatment of DMO in pregnant women with favourable outcomes both for the retina and the pregnancy [13, 16, 17]. Although a population-based cohort study from France suggested a high incidence of obstetric complications in pregnant women treated with intravitreal corticosteroids, this was thought to be related to maternal age and the underlying systemic comorbidities (e.g. diabetes, hypertension) driving the need for treatment in the first place [18].

Systemic corticosteroids are appropriate to use in pregnancy when indicated. Different steroids cross the placenta at varying rates, which influences how they are used in clinical practice. Non-fluorinated steroids such as prednisone and prednisolone (the active form of prednisone) cross the placenta only to a small extent (10–20%), while fluorinated steroids such as betamethasone or dexamethasone cross over completely (100%) [14, 15]. Prednisone and prednisolone are therefore used for systemic treatment of the mother, while dexamethasone is widely used for antenatal treatment of the fetus. No unfavourable outcomes have been reported at the lower doses of prednisolone used to treat chorioretinal disorders in pregnant women. While older animal studies showed that repeated maternal systemic betamethasone injections in pregnant sheep resulted in delayed fetal retinal maturation [19], these doses were greater than those now used in humans and are therefore less relevant to risk-benefit discussions with patients. However, close monitoring is important, as systemic corticosteroids can cause side effects such as hyperglycaemia that can be problematic in the third trimester.

Overall, if a pregnant woman receives corticosteroids as part of an established pre-pregnancy immunosuppression regimen, pregnancy alone should not be a reason to modify her treatment, but should prompt consideration of steroid-sparing agents, which can also be used in pregnancy [13, 14]. Ultimately, the risk of systemic side effects with topical, periocular, and intravitreal steroids is very low, and they should be used if indicated. As with all medications, appropriate counselling and documentation are essential. Each case should be addressed individually and the range of alternative therapeutic options discussed (Table 1) [13, 20].

**Table 1:** Topical, intravitreal, and systemic steroid agents and their safety profile in pregnancy.

Route	Corticosteroid agent	Indications	Safety profile in pregnancy	Adverse effects: systemic (maternal)	Adverse effects: systemic (fetus)	Adverse effects: ocular (maternal)
Topical	Prednisolone	Anterior segment inflammation, allergic eye disease, keratitis (some types)	Safe to use - low risk of systemic absorption	None	None	Exacerbation of herpetic infection, steroid-induced increase in intraocular pressure, and cataract
	Dexamethasone					
Intravitreal	Triamcinolone acetonide (dose not standardised, approx. 4 mg)	Diabetic macular oedema, cystoid macular oedema, posterior uveitis	No significant increase in systemic serum levels, so safe to use	Headaches (possibly in people who are predisposed to migraines)	Inadequate data	Steroid-induced increase in intraocular pressure, cataract, subconjunctival haemorrhage, corneal abrasion, inflammation, bleeding, retinal tear/ detachment, vitreous haemorrhage, endophthalmitis, temporary floaters
	Fluocinolone acetonide (slow-release tube 190 μg)		Insufficient data, but likely minimal change to systemic circulating level, so appropriate to use			
	Dexamethasone (slow-release implant 700 μg)		No significant increase in systemic serum levels, so safe to use			
Systemic	Dexamethasone	l Fetal lung maturation if preterm delivery anticipated	Crosses the placenta, but not metabolised by the placenta. Can be used in pregnancy for maternal indications, but alternative steroid preferred if possible	Hyperglycaemia, Bone changes, Gastro-oesophageal reflux	Preterm rupture of membranes	Cataract
	Prednisolone	Treatment of maternal systemic disorders, inflammatory chorioretinal conditions, anterior and posterior scleritis	Crosses the placenta but mostly metabolised by the placenta, so the steroid of choice for the treatment of maternal conditions			

## Treatment of steroid-related complications: intraocular pressure rise or steroid-induced glaucoma

Elevated IOP is an important side effect which can occur following the use of topical, intravitreal and rarely oral steroid medication. A meta-analysis suggests that this affects 32% (95% confidence interval (CI), 28.2–36.3) of individuals treated with intravitreal triamcinolone 4 mg, and 15% (95% CI, 9.2–24.3) of those treated with intravitreal dexamethasone 700 mcg (Ozurdex®) [21]. For topical steroids, an older study which defined the patterns of steroid response in normal adults following a 4-week course of dexamethasone 0.1% eyedrops suggested a minimal response (IOP rise of <6 mmHg) in 60%, intermediate response in 35% (6–15 mmHg) and high response (>15 mmHg) in 5% [22].

Steroid-induced IOP elevation tends to settle within 6 months of treatment cessation, but this may take up to 18 months and may require additional medical or surgical treatments [23]. It is unclear whether and how this risk changes in pregnancy. Persistently elevated IOP can lead to optic nerve damage and irreversible loss of vision, and the visual morbidity and downstream quality of life and economic effects this may engender may be significant. Although the physiological changes of pregnancy produce a small transient reduction in IOP (by up to 10% throughout pregnancy, before returning to baseline levels in the postpartum period) [24], this may not be sufficient to negate the need for treatment entirely.

The management of raised IOP tends to take a stepwise approach, starting with topical IOP-lowering eye drops (escalating from monotherapy up to quadruple therapy) and/or laser treatments to the trabecular meshwork in the iridocorneal angle. Surgery is often reserved for treatment-refractory and progressive cases. Managing IOP during pregnancy presents unique challenges, as many common IOP-lowering medications cause concern because of the potential risks to the developing fetus reported from use of the oral preparations of these same medications. Techniques such as punctal occlusion should be considered to further reduce the risk of systemic side effects.

Overall, the approach to IOP-lowering medications should be pragmatic. Several classes are used in non-pregnant patients, and the safety profile of each class in pregnancy is summarised in Table 2 and discussed below:

**Table 2:** Intraocular pressure-lowering medications and their safety profile in pregnancy.

Class of medication	Examples	Mechanism of action	Adverse effects—ocular	Adverse effects—systemic	Safety profile in pregnancy	Safety profile in Breastfeeding
Prostaglandin analogues	Topical Latanoprost, Bimatoprost, Travoprost	Selective agonist of prostaglandin F receptors increase aqueous humour outflow through the uveoscleral tract, acting on extracellular matrix and widening the spaces within the trabecular meshwork. Duration of action 24 h, dose once daily	Burning sensation, conjunctival hyperaemia, iris pigmentation, punctate epithelial keratopathy, dry eyes, iritis, herpes keratitis, uveitis	Flu-like syndrome, chest pain, allergic skin reaction, arthralgia, myalgia, and toxic epidermal necrolysis (very rare).	No known issues from topical treatments, safe to use	No known issues from topical treatments, safe to use
Beta-blockers	Topical Timolol, Betaxolol	Inhibition of β-receptors on the ciliary epithelium, reduction of aqueous humour production. Duration of action 12 h, dose once or twice daily	Burning sensation, conjunctival hyperaemia, dry eyes, periocular atopic dermatitis	Systemic absorption. Bradycardia, bronchospasm.	No known issues from topical treatments, safe to use	No known issues from topical treatments, safe to use
Alpha-2 adrenoceptor agonists	Topical Brimonidine	Reduction in aqueous humour formation and increase in uveoscleral outflow. Dose three times daily	Conjunctival hyperaemia, burning sensation, blurry vision	Headache	Limited data, probably compatible	Probably compatible
Carbonic anhydrase inhibitors	Acetazolamide (oral/IV), Dorzolamide, Brinzolamide (topical)	Suppresses aqueous humour secretion and increases aqueous humour outflow. Dose: acetazolamide orally 250–1000 mg daily, dorzolamide or brinzolamide three times daily	Topical agents: Conjunctival hyperaemia, burning sensation, allergic reaction, superficial punctate keratopathy	Acetazolamide: Fatigue, nausea, paraesthesia, metabolic acidosis, hyponatraemia, hypokalaemia, toxic epidermal necrolysis	No known issues from topical treatments, safe to use. Oral treatments can be used if no alternative	Both oral and topical preparations compatible with breastfeeding

*Alpha agonists* (example: brimonidine) are preferentially used in the first and second trimesters of pregnancy, and it has historically been recommended that using them in the late third trimester should be avoided due to theoretical concerns about the potential for central nervous system depression in the fetus [25–27]. Whilst one review advises against use in breastfeeding [28], ultimately, the systemic absorption from topical use is very small and is further reduced by punctal occlusion. Therefore, it is generally viewed that these can be used in breastfeeding as normal [29].

*Prostaglandin analogues* (examples: latanoprost, bimatoprost) in their non-topical form have historically caused concern during pregnancy due to the theoretical risk of uterine smooth muscle contraction increasing the possibility of miscarriage [30], but this has not been proven. The dose from eye drops is very low and does not warrant alteration in the usage of eye drops in pregnancy. UK clinical guidance has been updated to reassure pregnant women that topical latanoprost and bimatoprost can be used in pregnancy and breastfeeding as normal [31].

*Beta-blockers* (example: timolol) can cross the placental barrier, with oral preparations reported to potentially lead to fetal bradycardia, intrauterine growth restriction (IUGR), hypoglycaemia, and respiratory issues [32]. Oral beta-blockers are commonly used in pregnancy (e.g. labetalol for hypertension, bisoprolol for arrhythmias) and have not demonstrated any increased risk of major congenital anomalies [33]. While there is a potential dose–response effect of oral beta blockers on the risk of delivering a small-for-gestational-age baby (defined as birthweight <10th centile) [34], large registry studies suggest the true difference to be small (177 g), which is not felt to be clinically significant [35]. The systemic absorption of topical beta blockers is very small, so the dose to the fetus is significantly below that thought to cause any fetal issues. UK clinical guidance recommends that topical beta blockers can be used as normal in pregnancy [36].

*Carbonic anhydrase inhibitors (CAI)* (examples: acetazolamide, dorzolamide) may be used in pregnancy if no suitable alternatives are available. Oral preparations are often avoided in the first trimester, as oral CAIs have been associated with teratogenicity in animal studies, but at much higher equivalent doses than those which would be used in pregnancy [37]. Oral CAIs have been successfully used in pregnant women with idiopathic intracranial hypertension at much higher doses, without any convincing evidence for adverse effects, even when prescribed in the first trimester [38]. As the systemic absorption of topical CAI is very small, there is no clear evidence of harm, and these can be used as normal.

Non-pharmacologic options may also be considered. These can include selective laser trabeculoplasty (SLT), a non-invasive treatment modality that can be considered to reduce the IOP and/or medication burden in steroid-induced glaucoma [39]. Safety and efficacy in the non-pregnant population are well established, and treatment success is not limited by patient age [40]. While there are limited data on its use in the pregnant population, no reports of adverse maternal or fetal events have been published. Although the treatment response is often not sustained in the long term, this may suffice for the duration of the pregnancy [41]. However, it is important to note that SLT is not suitable for all forms of glaucoma, for example those resulting from a closed or abnormal iridocorneal angle. In treatment-refractory cases, surgical treatment options may need to be considered.

Ultimately, a multidisciplinary team approach (including glaucoma specialists, obstetric physicians/obstetricians, as well as medical retinal or uveitis specialists for the primary disease) and careful patient education regarding treatment compliance are key to achieving optimal outcomes for both mother and fetus. The development of patient-facing resources such as the NHS website in the UK can be helpful for providing information and reassurance [31, 36].

## Systemic immunosuppressants in pregnancy

Treatment of pregnant women with immunosuppressive medication is occasionally required, most commonly to control ocular inflammation secondary to autoimmune disorders or some cases of corneo-scleral transplantation. Many patients in this group will have been on these treatments before pregnancy, and the most suitable agent for maintaining disease control during pregnancy needs to be considered. For a few patients, treatment will need to be started during pregnancy. Adherence to immunosuppressive treatment is crucial to prevent autoimmune flare-ups or graft rejection, while close monitoring is essential throughout pregnancy. Counselling should include a multidisciplinary approach, with appropriate discussion about how pregnancy may affect the underlying condition and the potential effects of the medication on the fetus. The decision to taper or stop treatment due to theoretical risks should be made on a case-by-case basis, considering the patient’s medical history and potential outcomes.

Many immunosuppressive medications, including azathioprine, calcineurin inhibitors (ciclosporin A, tacrolimus), interferons, and TNF-alpha antagonists, are safe for use during pregnancy [5]. However, others, such as mycophenolate mofetil/mycophenolic acid and methotrexate, have proven to be embryotoxic [5]. Data on the safety of other agents (e.g. sirolimus, everolimus) are currently limited [14, 42, 43]. The immunosuppressive medications most commonly used to treat chorioretinal disease are described below.

### Azathioprine

Azathioprine is widely used to control ocular inflammation and can be used throughout pregnancy and breastfeeding. There is no evidence of increased risk of structural malformations. While there have been few reports of preterm delivery and fetal growth restriction, it is important to highlight that these women are often also on other medications and have systemic comorbidities such as autoimmune diseases, which are themselves associated with adverse pregnancy outcomes [42, 44, 45].

### Calcineurin inhibitors

Systemic administration of ciclosporin A may be used to treat autoimmune uveitis and corneal graft rejection. Topical ciclosporin (e.g. Ikervis®) is efficacious for dry eye syndrome and various immune corneal disorders. Repeated systemic administration can lead to complications such as nephrotoxicity and hypertension in the mother, and renal function and blood pressure should therefore be closely monitored after treatment initiation. Ciclosporin A crosses the placental barrier and may reach fetal blood concentrations of up to 50% of the concentration in maternal blood. There is no evidence of congenital malformations in children born to mothers taking either topical or systemic ciclosporin [22].

The use of tacrolimus in pregnancy is considered safe, and pregnant women may be newly commenced on treatment if required. Renal function and glucose levels should be monitored due to the risk of nephrotoxicity and hyperglycaemia [46]. Despite the high rates of placental transfer and *in utero* exposure, there are no data to suggest that the use of tacrolimus in pregnancy increases the risk of congenital malformations or fetal complications [42, 46, 47].

### Biologic agents

Anti-tumour necrosis factor (TNF) biologics are low risk in the first and second trimester of pregnancy, as there is no evidence of higher rates of teratogenicity or miscarriage [48]. Transplacental transfer of these agents can result in detectable serum levels in the infant several months after birth, which have historically raised concerns about the neonate’s immunological response to infections and vaccinations. As such, there has been hesitation in continuing biologics into the third trimester (after 28 weeks’ gestation), with some guidelines suggesting treatment cessation prior to this [48]. There is clearly a risk of a flare in the underlying condition with this approach, and this can have an adverse effect on the pregnancy and the fetus. In current practice, it is very common to continue biologics throughout the third trimester. Monoclonal Antibody Medications in Inflammatory Arthritis (MAMA) is a new randomised trial that will examine the impact of continuing or stopping biologics after 28 weeks of gestation in pregnant women with inflammatory arthritis [49]. The results will also provide useful information on pregnancy outcomes in women treated with biologics in general.

Different biologic agents cross the placenta at different rates. The Fc portion on antibodies such as adalimumab and infliximab facilitates placental transfer, but this does not seem to have an adverse effect on the fetus unless used together with azathioprine, a combination which may increase the neonatal infection rate [50]. In contrast, the absence of an Fc portion on certolizumab prevents placental transfer [50, 51]. It is therefore the only biologic agent licensed for use in pregnancy in Europe [52]. However, it has not been approved or tested for ophthalmic use. In practice, if women are well controlled on their biologic drug, this is not routinely changed to certolizumab before or during pregnancy.

Adalimumab and infliximab have been approved in the UK for the management of treatment-refractory or aggressive uveitis with sight-threatening complications. A large prospective study of adalimumab in pregnant women with Crohn’s disease or rheumatoid arthritis did not find an increased risk of birth defects, miscarriage, preterm delivery, pre and post-natal growth deficiency, serious or opportunistic infections, or malignancies [53]. Similarly, there have not been any reports of fetal issues with infliximab [54].

Rituximab may rarely be used in the management of treatment-refractory scleritis. Data on rituximab use in pregnancy is more limited due to less frequent use. There is a risk of B-cell depletion in a neonate born to a mother who received rituximab during pregnancy, but no significant adverse events have been reported [55]. Rituximab can be used in the first half of pregnancy, and is sometimes used in the second half for specific women where there is no appropriate alternative treatment.

Tocilizumab may be used as second-line therapy for thyroid eye disease. Clinical trials evaluating its use in patients with chorioretinal inflammatory diseases are ongoing. While safety data are limited, analyses from global safety databases have not found any increased risk of miscarriage or congenital malformations following maternal exposure to tocilizumab in the pre-conception phase or early pregnancy [56, 57]. Tocilizumab may be continued in pregnancy if required.

Live vaccines are not currently advised for babies born to mothers receiving biologics in the third trimester. There are two live vaccines in the UK neonatal vaccine schedule—the Bacille Calmette-Guérin (BCG) and rotavirus vaccines. BCG is offered in the neonatal period to babies at higher risk of tuberculosis (TB), such as those born into families with a higher background risk or those living in certain parts of the UK with high background rates of TB. It is advised that neonatal BCG vaccination is delayed for at least 6 months (12 months in women who took infliximab) due to the risk of inducing disseminated BCG [58]. Rotavirus infection typically causes diarrhoea and vomiting in infants. Some international guidelines advocate use of this vaccine as normal even in women who received biologics because of an absence of clear risks [59], however, UK guidelines currently advise omitting this entirely rather than postponing it [60]. All other vaccinations are safe and should be administered as normal to the infant [61].

### Mycophenolate mofetil

Mycophenolate mofetil is a teratogenic agent and should not be used during pregnancy. It has been associated with increased risk of spontaneous miscarriage and severe congenital malformations, and treatment should therefore be discontinued at least 4 weeks prior to conception [62, 63]. Women using mycophenolate mofetil should be transitioned to another immunosuppressive agent before attempting conception. Azathioprine is considered the agent of choice in such cases [64, 65]. Mycophenolate mofetil may sometimes be used after the first trimester, if no other alternatives are available.

### Methotrexate

Methotrexate is effective in the treatment of chronic non-infectious uveitis. It is contraindicated during pregnancy because of its teratogenic effect in humans, especially in the first trimester of pregnancy. Its use has been associated with increased incidence of spontaneous fetal loss and congenital defects [42]. Women of childbearing age requiring methotrexate should be advised on the teratogenic effects of these medications and the need for highly effective contraception during the treatment period. Women planning for pregnancy should transition away from methotrexate at least 3 months prior to conception [66]. Men were previously advised to do the same; however, they are now advised to continue treatment as usual [66] because an increasing body of evidence has not demonstrated any association between paternal methotrexate exposure and adverse pregnancy outcomes or congenital malformations [67].

### Janus kinase (JAK) inhibitors

Investigations into the use of JAK inhibitors for refractory non-infectious uveitis are ongoing. To date, they have been used to good effect in small cohorts of patients resistant to conventional treatments [68, 69]. While data on adverse pregnancy outcomes, including miscarriage, teratogenicity, and neonatal immunosuppression, are limited, their low molecular weight raises the possibility of transplacental transfer and therefore a risk for fetal development [63]. Currently, there is a lack of consensus around when to discontinue JAK inhibitors, with some guidelines recommending that this be done 2 weeks prior to conception, with others advising 2 months [62, 63].

Overall, systemic immunosuppressants are helpful in reducing the risk of recurrence and maintaining immune-mediated inflammatory disease quiescence. As a general rule, women of childbearing age on long-term immunosuppressants in the uveitis clinic are advised to use contraception, to inform their doctors if considering pregnancy, and are counselled on the risk of teratogenicity with certain drugs. It would be advisable for a comprehensive multidisciplinary team approach involving the appropriate healthcare professionals (uveitis specialist and obstetrician) and the patient to take place prior to conception. Pre-pregnancy counselling is a growing service offered by maternal medicine units to provide advice about plans for treatment options in advance of and during a pregnancy. An individualised plan regarding monitoring and treatment regimens is essential, including tapering or switching of potentially teratogenic medications with a view to optimise both pregnancy and visual outcomes [14, 42].

Local treatment options, such as corticosteroid intravitreal implants, while not necessarily providing adequate treatment in the long term, may be able to provide sufficient temporary local immunosuppression to last throughout the pregnancy period in some types of uveitis without associated systemic disease.

## Anti-VEGF agents in pregnancy

Intravitreal anti-VEGF agents such as aflibercept (Eylea®), ranibizumab (Lucentis®), bevacizumab (Avastin®) and increasingly faricimab (Vabysmo®) are commonly used in non-pregnant patients for the management of several chorioretinal diseases such as macular neovascularisation (MNV) (related to age-related macular degeneration (AMD), myopia, inflammatory chorioretinal diseases or other secondary causes), DMO, cystoid macular oedema related to retinal vein occlusion, and radiation maculopathy. However, some ophthalmologists may be hesitant to prescribe them to pregnant women because VEGF is required in modulating placental and embryonic vascular development, meaning that systemic absorption of anti-VEGF could theoretically affect the pregnancy. This has contributed to a paucity of data on maternal and fetal outcomes [70, 71].

The choice of anti-VEGF agent often depends on the indication for treatment. For example, in the UK, ranibizumab, aflibercept and faricimab are licensed for the treatment of macular oedema secondary to diabetes and retinal vein occlusion. Biosimilars of ranibizumab have demonstrated real-world safety and efficacy in this regard, and are similarly licensed for use in the UK [72]. In terms of treating MNV, both aflibercept and ranibizumab are licensed for myopic MNV, whereas off-label treatment with bevacizumab would be offered for MNV secondary to other causes, such as central serous chorioretinopathy or pseudoxanthoma elasticum. Another important factor to consider is that bevacizumab is not formulated or licensed for intravitreal use for any of these indications (although bevacizumab gamma has recently been approved for use in neovascular AMD, which is not relevant to this age group), and so patients should be counselled regarding the risks of off-label use [73].

Systemic absorption of the intravitreal anti-VEGF agent is an additional factor to consider in pregnant patients. There are insufficient data to determine which anti-VEGF agent is safest in pregnancy. Ranibizumab is likely to be preferable since, according to pharmacokinetic studies, it has the lowest systemic absorption and shortest half-life, while aflibercept would theoretically confer the highest risk in humans [70, 74, 75]. In addition, animal studies have not demonstrated any adverse pregnancy or fetal outcomes for ranibizumab, in contrast to systemic bevacizumab (which had an embryotoxic and teratogenic effect, but at much higher doses than that achieved by intravitreal administration) and aflibercept, which induced embryotoxicity [73, 75].

A systematic review of cases treated with intravitreal anti-VEGF in the literature identified 41 women who were treated over the course of 42 separate pregnancies [9]. Bevacizumab (*n* = 22, 54%) and ranibizumab (*n* = 17, 41%) were administered more frequently than aflibercept (*n* = 2, 5%), although up to 40% (*n* = 6) were treated early in the first trimester before their pregnancy was recognised, meaning that pregnancy status was not a consideration for anti-VEGF agent in many cases. In terms of pregnancy outcomes, the majority (*n* = 34, 81%) resulted in live births, five of which were complicated by pre-eclampsia, premature delivery, and/or IUGR, all in women with significant risk factors for these adverse pregnancy outcomes, such as pre-existing diabetes. There were three stillbirths in women with complex obstetric histories, such as recurrent miscarriages or stillbirths. There were five early-term miscarriages, which comprised four women with additional risk factors for poor pregnancy outcomes, and one very early pregnancy loss at four weeks’ gestation. Miscarriage in early pregnancy is common, so a direct association between obstetric outcomes and anti-VEGF treatment cannot be drawn. Published data on neonatal complications were limited.

Standard anti-VEGF treatment regimens in the non-pregnant population often involve a loading course of three to five injections followed by maintenance treatment with extended treatment intervals. It would be reasonable to consider treatment on a *pro re nata* (PRN, or ‘as required’) basis rather than a standard dosing schedule in pregnant patients, to minimise the number of injections required during pregnancy, but only if this is not thought to compromise the overall therapeutic benefit. Most patients in the aforementioned systematic review only received one to two injections during pregnancy with a good treatment effect [9].

Given the limited safety data on the use of anti-VEGF agents in pregnant women, we would recommend a multidisciplinary team approach to include obstetric physicians/obstetricians, ophthalmologists, and any relevant specialist physicians involved in their medical care when deciding on whether to proceed with anti-VEGF treatment. Patients should be appropriately counselled and actively involved in the decision-making process, with opportunities offered to have a detailed discussion of the potential risks, benefits, and alternatives with a knowledgeable clinical team. This may include observation without treatment, or alternatives such as Ozurdex® for conditions such as diabetic macular oedema or cystoid macular oedema associated with retinal vein occlusions. However, Ozurdex® is less suitable for steroid responders, and confers a risk of cataract formation with repeated treatments, although development of a visually significant cataract with a single implant is unusual in uncomplicated injections [76]. For other sight-threatening conditions, such as MNV, where there are no other therapeutic options aside from anti-VEGF agents to preserve vision, multidisciplinary team discussion becomes even more essential.

More research is needed on the safety of anti-VEGF treatment in the peri-conception period, and the time interval for which pregnancy should be avoided post-treatment. There are insufficient safety data to recommend an optimal time point at which anti-VEGF injections can be safely administered in pregnancy, although delaying until after 12 weeks could be considered (as with other medications) if this was not felt to be detrimental to the trajectory of the eye condition. The anti-VEGF agents are summarised in Table 3.

**Table 3:** Anti-vascular endothelial growth factor (VEGF) agents and their safety profile in pregnancy.

Anti-VEGF agent	Indication for treatment	Target	Molecular weight	Dose per injection	Adverse effects	Safety profile in pregnancy—teratogenicity
Ranibizumab (Lucentis® and biosimilars)	Macular neovascularisation due to myopia, uveitis/inflammation etc.	VEGFA	48 kDa	0.5 mg	Ocular: Subconjunctival haemorrhage, inflammation, bleeding, retinal tear/detachment, vitreous haemorrhage, cataract, increased intraocular pressure, endophthalmitis, corneal abrasion, occlusive retinal vasculitis (rare)	Preferred if anti-VEGF required. Lowest risk (lowest systemic absorption, shortest half-life), no reported toxicity (animals)
Aflibercept (Eylea®)		VEGF A, VEGF B, PDGF	97–115 kDa	2 mg		Avoid due to theoretically higher risk of systemic absorption
Faricimab (Vabysmo®)	Diabetic macular oedema	VEGF A, Ang-2	150 kDa	6 mg		Avoid due to inadequate data
Bevacizumab (Avastin®)	Cystoid macular oedema due to retinal vein occlusion	VEGFA	149 kDa	1.25 mg		Systemic use associated with embryotoxicity Not licensed for intravitreal use (except in neovascular AMD)—available off-label
Brolucizumab (Beovu®)		VEGF A	26 kDa	6 mg		Avoid due to inadequate data

## Modes of childbirth in pregnant women with chorioretinal disease

Valsalva-like manoeuvres during the second stage of labour increase intra-abdominal pressure. Historically, there has been some concern that this might lead to a rise in IOP [77] and contribute to retinal conditions such as rhegmatogenous retinal detachment (RRD) or subretinal haemorrhage, which may therefore influence the recommended mode of delivery [78]. However, there is no clear evidence for this.

Previous surveys in Canada and Iran found that obstetricians were more likely to recommend operative delivery (caesarean section or assisted vaginal delivery to shorten the second stage of labour) in pregnant women who were high myopes and/or had other risk factors for RRD, in contrast to ophthalmologists who tended to feel spontaneous vaginal delivery was acceptable and appropriate [79, 80]. Importantly, there is no evidence to suggest that vaginal delivery in women with retinal lesions predisposing to RRD or a previous history of retinal detachment confers a higher risk of re-detachment [81, 82]. Caesarean delivery is therefore not mandated in this patient group.

There have been similar concerns about vaginal delivery inducing subretinal haemorrhage in patients with conditions related to breaks in Bruch’s membrane. An example of this would be angioid streaks secondary to pseudoxanthoma elasticum, but a case series did not find any evidence for an increased risk of subretinal haemorrhage in these patients [83]. There is only a single case report of post-partum subretinal haemorrhage from lacquer cracks secondary to high myopia in the literature [84], supporting advice that lacquer cracks and/or myopia should not necessarily be a contraindication to vaginal delivery. Active MNV has previously been thought to be an ophthalmic indication for caesarean section due to this risk, and has previously been recommended as such [85, 86]. However, research on this is lacking. A multidisciplinary team discussion involving the patient and the relevant specialists is advised to weigh up these theoretical risks and additional obstetric considerations [87].

## Conclusion

We have summarised the available evidence on several key areas of ophthalmic management in pregnant women, including a discussion on complex and potentially controversial topics such as treatment with intravitreal anti-VEGF agents in pregnancy, IOP-lowering medications, and considerations on mode of delivery in pregnant women with chorioretinal disease. Overall, the published literature on these topics tends towards relatively low-quality studies (in terms of study design and sample size), limited data on treatment outcomes and safety in pregnant women (rather than animal models), and old data from several decades ago, when treatment options were more limited. Decision-making regarding ophthalmic treatments therefore needs to balance the theoretical risk of drug exposure on fetal health versus the risk of untreated chorioretinal disease on maternal vision, bearing in mind that while the safety data in pregnancy may be limited, many of these treatments have proven visual benefits that are often supported by level one evidence from randomised controlled trials. In general, the systemic absorption from topical, periocular and intravitreal medications is typically very low and a theoretical risk of side effects should not prevent pregnant women from accessing necessary medications. As such, pregnancy should not be assumed to prevent the use of normal ophthalmic treatments in sight-threatening chorioretinal pathology, and chorioretinal pathology is rarely an indication for caesarean delivery. Key principles of managing these complex cases include pre-pregnancy counselling for pre-existing ophthalmic conditions and multidisciplinary team discussions comprising ophthalmologists, obstetricians, and obstetric physicians. It is essential for clinicians to establish these important relationships in order to optimise patient care, and to support pregnant women in weighing up the risks and benefits of treatment versus not having treatment (in terms of effects on the fetus as well as their eyesight) to enable them to make a decision that is right for their individual circumstances within a shared decision-making process.

## Supplementary information

Supplementary S1: PubMed search strategy for the narrative review.

## Data Availability

Data sharing is not applicable to this article as no datasets were generated or analysed during the current study.
