# Supplementary material for: Management of uveitic and chorioretinal conditions in pregnancy
Source: Eye (Lond). 2025 Nov 7;40(1):24–33. doi: 10.1038/s41433-025-04058-9 (PMC12764948; doi:10.1038/s41433-025-04058-9)
Supplement: Supplementary file 1 — Supplementary S1: PubMed search strategy for the narrative review. [file 41433_2025_4058_MOESM1_ESM.docx]

**Supplementary Materials**

**S1: PubMed search strategy for the narrative review.**

Combining key concept #1 with each of key concept #2:

| 1. **Pregnancy** |  | (Pregnancy[Mesh] OR pregnan*[tiab]) |
| --- | --- | --- |
| 1. **Management** | **Corticosteroids** | ((steroid*[tiab] OR corticosteroid*[tiab] OR prednisone[tiab] OR prednisolone[tiab] OR dexamethasone[tiab] OR triamcinolone[tiab] OR betamethasone[tiab])) AND (("Intravitreal Injections"[Mesh] OR "Administration, Oral"[Mesh] OR "eye drop"[tiab] OR intravitreal[tiab] OR periocular[tiab])) |
|  | **Intraocular pressure lowering treatment** | ("Intraocular Pressure"[Mesh] OR "Ocular Hypertension"[Mesh] OR "Glaucoma"[Mesh] OR "intraocular pressure"[tiab] OR IOP[tiab] OR "ocular hypertension"[tiab]) |
|  | **Systemic immunosuppressants** | ("Immunosuppressive Agents"[Mesh] OR immunosuppress* OR azathioprine[tiab] OR cyclosporin*[tiab] OR tacrolimus[tiab] OR biologic*[tiab] OR adalimumab[tiab] OR infliximab[tiab] rituximab[tiab] OR tocilizumab[tiab] OR mycophenolate[tiab] OR methotrexate [tiab] “JAK inhibitor”[tiab]) |
|  | **Intravitreal anti-vascular endothelial growth factor (anti-VEGF)** | (“anti-vascular endothelial growth factor" OR anti-VEGF[tiab] OR bevacizumab[tiab] OR ranibizumab[tiab] OR aflibercept[tiab] OR pegaptanib[tiab] OR conbercept[tiab] OR brolucizumab[tiab] OR faricimab[tiab] OR “intravitreal injection” [tiab]) |

Combining concepts for delivery and key chorioretinal disease

("Delivery, Obstetric"[Mesh] OR childbirth*[tiab] OR “mode of delivery”[tiab])

AND

("Choroidal Neovascularization"[Mesh] OR "Retinal Detachment"[Mesh] OR "Retinal Diseases"[Mesh] OR choroidal neovasculari*[tiab] OR CNV[tiab] OR retinal detachment[tiab])
